# Rank Pooling Approach for Wearable Sensor-Based ADLs Recognition

**DOI:** 10.3390/s20123463

**Published:** 2020-06-19

**Authors:** Muhammad Adeel Nisar, Kimiaki Shirahama, Frédéric Li, Xinyu Huang, Marcin Grzegorzek

**Affiliations:** 1 Institute of Medical Informatics, University of Lübeck, Ratzeburger Allee 160, 23562 Lübeck, Germany; li@imi.uni-luebeck.de (F.L.); huang@imi.uni-luebeck.de (X.H.); grzegorzek@imi.uni-luebeck.de (M.G.); 2 Department of Informatics, Kindai University, 3-4-1 Kowakae, Higashiosaka City, Osaka 577-8502, Japan; shirahama@info.kindai.ac.jp

**Keywords:** rank pooling, human activity recognition, activities of daily life, atomic activities, composite activities

## Abstract

This paper addresses wearable-based recognition of Activities of Daily Living (ADLs) which are composed of several repetitive and concurrent short movements having temporal dependencies. It is improbable to directly use sensor data to recognize these long-term *composite activities* because two examples (data sequences) of the same ADL result in largely diverse sensory data. However, they may be similar in terms of more semantic and meaningful short-term *atomic actions*. Therefore, we propose a two-level hierarchical model for recognition of ADLs. Firstly, atomic activities are detected and their probabilistic scores are generated at the lower level. Secondly, we deal with the temporal transitions of atomic activities using a temporal pooling method, *rank pooling*. This enables us to encode the ordering of probabilistic scores for atomic activities at the higher level of our model. Rank pooling leads to a 5–13% improvement in results as compared to the other popularly used techniques. We also produce a large dataset of 61 atomic and 7 composite activities for our experiments.

## 1. Introduction

The advancement in health sciences has increased the median age of human and caused an increase in the number of elderly people. While it is good news overall, it raises other questions like whether the elderly people can maintain their quality of living independently or would need human resources to look after them. Medical doctors actually use Activities of Daily Living (ADLs) like dressing up, cleaning a room, or preparing a meal, to evaluate an elderly’s ability for independent living [1,2]. Thus, a system that can continuously recognize ADLs based on wearable devices, is beneficial for detecting their health problems and supporting healthcare.

An ADL is usually a long term and complex activity that is made up of the sequence of short-term actions. For example, preparing a meal can be made up of cutting the food, opening or closing the door of the refrigerator, stirring in the utensils. We refer to these long-term activities (i.e., ADLs) as *composite activities* and the underlying short-term actions as *atomic activities*. Wearable devices can be used to detect such activities in an unobtrusive way using motion sensors embedded on these devices [3,4,5]. In most cases, it is impractical to directly use raw sensor data to recognize composite activities [6] because raw data is cluttered with noise and meaningless components. The sensors may also yield different data streams for multiple examples (i.e., data sequences, in further reading, the term *example* will refer to a data sequence collected by the sensors) of the same activity even when performed by the same person. One main reason is that the person can change the order of atomic activities to accomplish the composite activity. Therefore, it is required to extract meaningful features from sensory data to recognize the composite activities.

**Figure 1:**
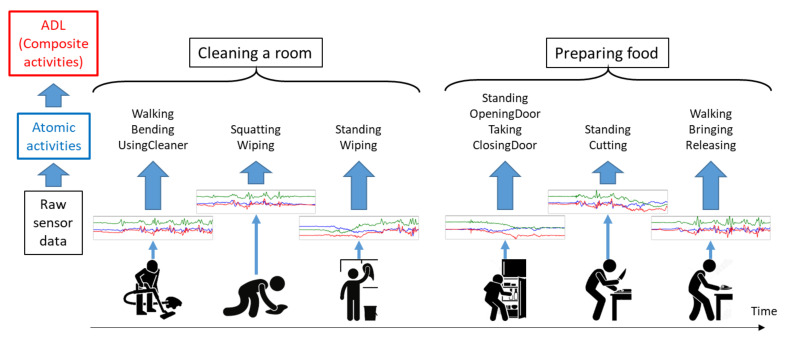
Hierarchical model for Activities of Daily Living (ADL) recognition. This figure depicts two examples of ADLs or composite activities, cleaning a room and preparing food. Each of the composite activities consists of several atomic activities like Bending, UsingCleaner, Squatting, Wiping, Opening Door and Cutting Food. Our hierarchical model uses raw sensory data obtained from wearable devices at its first level to detect the atomic activities performed within a short interval of time. The second level of our model recognizes composite activities based on the probabilistic scores of atomic activities.

Atomic activities can be considered as meaningful features for this purpose for two reasons. Firstly, despite personal habits, atomic activities having short durations are relatively easy to recognize using sensory data. Secondly, the example of the same composite activity generates different sensory data, but it has smaller diversity in terms of atomic activities, for example, the composite activity, cleaning a room can be characterized by the combination of atomic activities as depicted in Figure 1. When cleaning a room, a person is mainly walking, bending, and squatting to clean floors using a vacuum cleaner and a floor cloth, and sometimes standing to clean surfaces like windows and tables.

Thus, a composite activity may produce significantly different raw sensory data but they are assumed to be similar in terms of atomic activities. Hence, it is pertinent to recognize a composite activity on the basis of the atomic activities. It motivates us to propose a two-level recognition model in which atomic activities are detected at the first level and then the composite activities are recognized at the next level.

In order to recognize composite activities, we need to handle temporal relationships among underlying atomic activities. Hidden Markov Models (HMM) and Long Short Term Memory (LSTM) are popular techniques to deal with such temporal patterns. HMM are useful only when the classification target has a typical temporal relation and a clear structure [7], whereas LSTM can only be successfully used if we have an extremely large amount of data. Another technique used in event detection through video data is *pooling*. The temporal patterns of many concepts are involved to detect an event. Thus, a large amount of data describes one event. In order to compress this data, researchers have used pooling techniques. Some pooling techniques like max and average pooling have been found to be useful for activity recognition [8,9]. These techniques are usually applied to the recognition scores of atomic activities for the construction of feature vectors to characterize composite activities. The max and average pooling techniques work well but they are too simple and incapable of handling the temporal patterns within the example of a composite activity.

Thus, to solve this problem, we propose another pooling technique, *rank pooling*, that models the evolution and dynamics of a composite activity. Rank pooling is a temporal pooling method based on unsupervised learning. It accumulates the pertinent information during the execution of a composite activity by fitting a learning-to-rank model. Then, parameters of the learned model are used as the new features of the composite activity. Rank pooling not only maintains the temporal ordering of underlying activities but also performs well in a situation when there is no clear temporal relationship among these sequences. Rank pooling has been successfully used to recognize actions in video data [10]. We extend this technique to recognize composite activities using sensory data. We start by recognizing atomic activities and then find their temporal ordering by training a linear ranking model [11] on the recognition scores of atomic activities. The parameters of the linear ranking model encode the evolution of atomic activities that make up a particular composite activity. The ranking models trained on different examples of the same composite activity are expected to be similar. Therefore, we propose to use the parameters of the ranking models as characteristic features for the classification of composite activities.

Figure 1 shows an overview of our proposed two-level hierarchical model based on rank pooling. The lower level of our model recognizes atomic activities like sitting, standing, walking, standing up, squatting, opening and closing door. The higher level of our recognition model recognizes composite activities like cleaning room and preparing food. Atomic activities are detected by a codebook approach [12] which outputs the probabilistic scores of each of atomic activities. Rank pooling is then used to construct feature vectors for composite activities using these probabilistic scores. Classifiers trained on feature vectors obtained from rank pooling turn out to be very effective for distinguishing composite activities. We evaluate our results obtained by rank pooling while comparing them with the other pooling techniques like average and max pooling and also with HMM and LSTM. We found that rank pooling yields an accuracy 5 to 13% higher than the other techniques. In this paper, we also introduce a dataset of atomic and composite activities collected using wearable devices at our pattern recognition lab. We performed all of our experiments using this dataset.

This paper offers two main contributions:

- We use rank pooling for sensor based data to recognize 7 composite activities.
- We produce a large dataset that contains 9029 examples of 61 atomic activities and 890 examples of 7 composite activities. This dataset is collected within the Cognitive Village Project supported by the German Federal Ministry of Education and Research (BMBF), and named CogAge dataset for the sake of simplicity.

All research contents (CogAge dataset, codes, and trained models) are made available at the webpage: https://www.info.kindai.ac.jp/~shirahama/rank_pooling (in the Supplementary Materials) to help other researchers reproduce our findings.

This paper is organized as follows: Section 2 presents an overview of highly relevant existing research work regarding human activity recognition. Section 3 describes the details of our proposed methods to detect atomic and composite activities. Section 4 presents the experiments carried out on CogAge dataset and the results to evaluate of our proposed approach while comparing it with other state-of-the-art techniques. Section 5 provides discussions to use our proposed system in real-world settings for monitoring the ADLs using an online version of our system. Finally, Section 6 concludes the paper and presents potential future works regarding human activity recognition. Apart from the main body of this paper, Appendix A details the sensor modalities and data acquisition process. Our webpage shows detailed results like confusion matrices for recognition of 7 composite activities, and the details of our atomic activity recognition method which is not the main focus of this paper.

## 2. Related Work

In this section we discuss the existing research work that is highly relevant to our contribution. We start by mentioning hierarchical activity recognition models and then discuss about approaches to capture temporal patterns of activities. We also mention some deep-learning approaches for activity recognition. In the end, we relate the setup of our devices with similar existing approaches.

A long term activity exhibits a hierarchy of intrinsic actions. The research has shown that hierarchical models are necessary for accurate activity recognition [13,14]. In [15] the authors have presented a multilevel discriminative model to detect composite activities. Their hierarchical partonomy based approach shows improvements in recognition of composite activities and atomic activities (which are called activity events in [15]) compared to the non-hierarchical approach. However, they use a predefined sequence of atomic activities during the execution of a composite activity. The composite and fine-grained activities related to cooking are detected in [16] using video data. The authors have recognized fine-grained activities like peeling and washing, using pose-based, hand-centric, and holistic approaches. Firstly, they have detected the movements and positions of hands, and the shape of objects like knife and vegetable. Then they recognize composite activities based on the fine-grained activities. Their work produces a large dataset of kitchen related activities. The authors in [17] focus on recognition of complex activities based on a limited amount of labeled data by proposing an attribute-based learning. Their approach detects semantic attributes (primitive actions) in the existing data of composite activities and recognizes new activities on the basis of these attributes along with the characteristic features. A hierarchical model is presented in [18] to detect human activities in the wild using a smartphone accelerometer. This approach reduces the energy consumption and users do not need to wear a lot of sensors. The authors in [19] present a multimodal and multi-positional body sensing approach for recognizing ADLs. They use a two level classification technique to classify 21 different activities. They use wearable devices on multiple positions on the body for context awareness during the execution of an activity. Moreover, they use Bluetooth beacons for location awareness to increase the accuracy of their results. In [20], authors present an approach to automatically construct mid-level features using Deep Belief Networks. These features contribute to recognize well-structured human activities in an exercise dataset.

Compared to the aforementioned approaches, we present a more generic and a hierarchical approach that recognizes composite and the underlying atomic activities using multimodal sensory data obtained from three unobtrusive wearable devices. The fusion of multi-positional and multiple modalities of sensory data provides higher accuracy for our targeted 61 atomic and 7 composite activities. The automatic construction of atomic activities as described in [20] is data dependent and can be useful in a specific domain of activities. On the other hand, we manually define our atomic activities to make our hierarchical approach more general. We believe that our approach, where a large number of atomic activities cover numerous variational movements, is applicable for many composite activities other than the ones in this paper.

Generative models, particularly Hidden Markov Models (HMMs), encode temporal information and have largely been used to recognize long-term activities [21,22]. Hybrid approaches have also been used where a discriminative model like Support Vector Machine (SVM) or Artificial Neural Network (ANN) obtain posterior probabilities of actions and an HMM is employed to capture the temporal dynamics of the composite activities [23]. Another generative method is naive Bayesian network that can also deal with temporal relationships of activities. This approach has been used to encode temporal relationships in order to detect ADLs in home settings [24]. A layered HMM approach is proposed to detect human activities in an office environment [25] using multimodal data. It exploits the ability of HMM to handle different levels of abstraction and their corresponding time granularities. HMMs have also been used to recognize daily routine activities such as commuting and other office work tasks using unsupervised learning methods [26]. The authors in [27] present an HMM based activity recognition where sensors are placed not only on user’s body but also on the object used, in order to minimize the overhead of labeling and feature selection. Another popular model is Conditional Random Fields (CRFs) that capture sequential characteristics of long-term activities in a discriminative way [19]. In [15] the authors have classified the composite activities using the combination of joint boosting and CRFs. Many approaches from the field of deep learning have also been employed in the domain of human activity recognition [28,29,30,31,32] and produced very good results.

However, an HMM can be used only for recognizing activities that have a typical sequence of underlying actions [22]. In addition, it is incapable of capturing long-range or transitive dependencies of observations due to its very strict independence assumptions on observations. Furthermore, without a significant amount of training data, the HMM may not be able to recognize all of the possible observation sequences that can be consistent with a particular activity [21]. In contrast to HMMs, CRFs relax the independence assumptions on observations and can capture their long-rage dependencies. However, the biggest disadvantage of CRFs is their high computational complexity when there are a large amount of training data, and when there are many states having complex connectivities [33]. Although deep learning methods produce good results, they need a huge amount of training data and tune a large number of hyper-parameters. Finally, for all of HMMs, CRFs, and deep learning methods, finding a model structure that leads to accurate recognition needs a laborious trial-and-error process or significant amount of background knowledge on a problem.

Compared to the aforementioned methods, we propose a sensor-based activity recognition method using rank pooling, which has been originally developed to recognize human activities in video data [10]. A ranking function is learned that satisfies the sequential order of low-level atomic activities, in which a particular high-level composite activity is performed. The temporal evolution of atomic activities is learned by employing point-wise ranking functions [11]. One main advantage of rank pooling over the above-mentioned methods is that it only involves one hyper-parameter, that is, the penalty parameter for violating the sequential order of atomic activities. Thus, our method based on rank pooling can be easily applied to many problems without relying on any laborious trial-and-error process or much background knowledge.

The ubiquitous devices are considered unobtrusive for monitoring and recognizing ADLs and an abundant amount of work focuses on design and operations of these ubiquitous technologies in home settings [34,35]. The use of wearable sensors for such in-home settings has been experimented in [36] and we also use similar wearable devices for our activity recognition system.

## 3. Methodology Description

In this section, we describe our two-level hierarchical model for recognition of ADLs. The first level uses a codebook approach to generate recognition scores of atomic activities, and the second level recognizes ADLs by performing rank pooling on these scores.

### 3.1. Atomic Activity Recognition

First of all, assume that our atomic activity recognition method aims to recognize *M* atomic activities using *S* sensors, each of which forms a multi-dimensional sequence. Under this setting, our method consists of the two main phases, “feature extraction” and “model training/test”. For each of *S* sensors, the former extracts a feature that represents the characteristics of the corresponding sequence. Then, features for all the *S* sensors are fused into a single high-dimensional feature. This way, diverse examples for atomic activities in training data are converted into high-dimensional features. In the model training/test phase, a recognition model is built on those high-dimensional features. Afterwards, given sequences obtained from *S* sensors for an unknown example (i.e., test datum), the model is used to compute atomic scores that individually indicate the probability of an atomic activity in the example. Since atomic activity recognition is not the main focus of this paper, the feature extraction and model training/test phases are briefly described below. Please refer to the website of this paper for details of our atomic activity recognition method.

**Figure 2:**
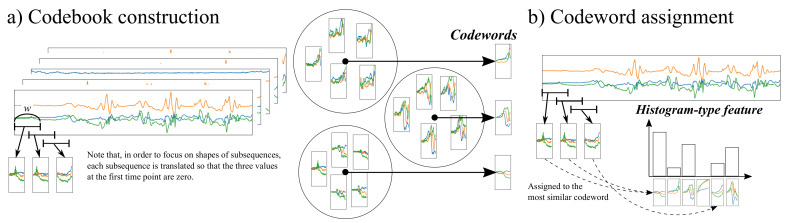
An overview of our codebook-based feature extraction phase: (**a**) illustrates the codebook construction step where subsequences collected from a large number of sequences are grouped into clusters, and the center of each cluster is regarded as a codeword; (**b**) depicts the codeword assignment step where each of subsequences in a sequence is assigned to the most similar codeword. As a result, the sequence is represented as a histogram-type feature indicating the frequency of each codeword.

Figure 2 illustrates the feature extraction phase based on the codebook approach, which extracts a histogram-type feature representing frequencies of characteristic subsequences, called codewords, in a sequence [12]. As shown in Figure 2, the codebook approach consists of two main steps. The first step, termed codebook construction in Figure 2a, adopts a sliding window approach to collect subsequences from a large number of sequences. These subsequences are then grouped into clusters of similar ones using *k*-means clustering. A codebook is constructed as a set of codewords each being the center of a cluster. The second step, named codeword assignment in Figure 2b, extracts the feature of a sequence by assigning each subsequence to the most similar codeword. Thus, the resulting feature is a histogram representing the frequency of each codeword in the sequence.

There are three notes about the feature extraction phase. The first is about how to measure the similarity between two subsequences. Let us assume that a sensor produces a *d*-dimensional sequence and subsequences are collected using a sliding window of length *w*. This means that each subsequence contains d×w values and can be regarded as a dw-dimensional vector. In Figure 2a, the sensor generates 3-dimensonal sequences (d=3), so each subsequence is represented as a 3w-dimensional vector. In addition, in order to focus on shapes of subsequences, each subsequence is translated so that the *d* values at the first time point are zero. Under this setting, the similarity between two subsequences is measured as the Euclidean distance between their dw-dimensional vector representations. The second note is based on the fact that the translation described above loses the overall distribution of values in a sequence. Thus, the codebook approach in [12] is extended by adding dimensions capturing such a distribution to the original codebook-based feature. Please refer to the website of this paper for details of this extension. The last note is regarding how to fuse features extracted from sequences for *S* sensors. We use a simple but effective approach, called early fusion, which just concatenates features for *S* sensors into a high-dimensional feature [12].

In the model training/test phase, for each of *M* atomic activities, the one-versus-all approach is used to build a model that distinguishes examples of this atomic activity from those of the other atomic activities. Since each example is represented by a high-dimensional feature through the fusion of component features for *S* sensors, a Support Vector Machine (SVM) is employed as a model because of its effectiveness for high-dimensional data [12]. It can be thought that the high-dimensional feature of each example indicates the location of this example in the high-dimensional space. The SVM is trained to output a scoring value between 0 and 1 based on the distance between the example and the classification boundary in the high-dimensional space. This scoring value is nothing except for the atomic score, and the larger it is, the more likely the example is to include the atomic activity. Finally, *M* SVMs are trained to output *M* atomic scores for the example. The online use of these SVMs is straightforward, that is, they are used to compute atomic scores for sequences acquired from *S* sensors in a certain time interval.

### 3.2. Composite Activity Recognition

**Figure 3:**
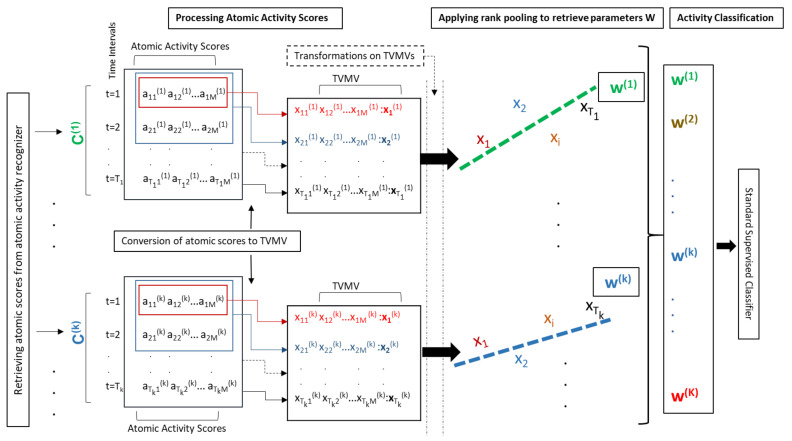
An overview of our composite activity recognition using rank pooling. The figure shows two examples $C^{\left(1\right)}$ and $C^{\left(k\right)}$ of a composite activity. Our atomic activity recognizer computes atomic scores in regular time intervals ($t=0\to T_{k}$) during the execution of each example of the composite activity. These atomic scores are converted to Time Varying Mean Vectors (TVMVs) (denoted by $x_{t}$). After applying non-linear transformations, we implement rank pooling on $x_{t}^{\left(k\right)}$s to compute the parameters $w^{\left(k\right)}$ of a ranking function $\Psi^{\left(k\right)}$. These parameters are used as a feature vector to represent an instance of $C^{\left(k\right)}$. Such feature vectors are generated for all instances in the training dataset. We use a Support Vector Machine (SVM) classifier to classify the composite activities.

We aim at classifying *N* composite activities using the atomic scores of *M* atomic activities that we computed using the method described in the previous section. Assume that we have *K* instances of *N* composite activities where each instance has its own length. Mathematically, the *k*th instance of a composite activity $C^{\left(k\right)}$ (1≤k≤K) has a length of $T_{k}$ time points. Figure 3 illustrates our composite activity recognition method where we first extract the atomic scores for $C^{\left(k\right)}$, which is described as,

(1) $$C^{\left(k\right)}=\left(a_{1}^{\left(k\right)},a_{2}^{\left(k\right)},\cdots ,a_{T_{k}}^{\left(k\right)}\right),$$

where each $a_{i}^{\left(k\right)}\in R^{M}$ denotes the vector of *M* atomic scores at a time point *i* ($1\leq i\leq T_{k}$) for $C^{\left(k\right)}$. Each $a_{i}^{\left(k\right)}$ can be further described as,

(2) $$a_{i}^{\left(k\right)}=\left(a_{i1}^{\left(k\right)},a_{i2}^{\left(k\right)},\cdots ,a_{ij}^{\left(k\right)}\cdots ,a_{iM}^{\left(k\right)}\right),$$

where $a_{ij}^{\left(k\right)}$ represents the atomic score of *j*th atomic activity (1≤j≤M), computed at time point *i* for $C^{\left(k\right)}$. In addition, the value range of $a_{ij}^{\left(k\right)}$ is between 0 and 1.

All $a_{i}^{\left(k\right)}$ vectors are shown according to their temporal ordering as we assume that the relative ordering of atomic activities is relatively preserved in the formation of a composite activity. So far, we have defined a composite activity in terms of its *k*th instance $C^{\left(k\right)}$ because we want to emphasize that each instance may have its own length. However, the use of similar terms without the *superscript* (k) refers to a composite activity in general instead of its specific instance i.e., $a_{t}^{\left(k\right)}$ belongs to *k*th instance whereas $a_{t}$ refers to vector of atomic scores for any instance of a composite activity. Before describing the method of capturing the temporal evolution or dynamics of atomic activities, we concede that atomic scores are expected to have a high degree of variability and noise. Using those scores directly might have some disadvantages. For instance, the noise may lead to the erroneous results. In addition, the time-independent vectors of atomic scores $a_{i}$ are weakly connected to the consecutive score vectors (i.e., $a_{i-1}$ and $a_{i+1}$) resulting in the sudden variations in atomic scores. This weak correlation may also cause an improper learning of the dynamics of atomic activities.

Thus, to reduce the effect of noise and abrupt variations in atomic scores, and to strengthen the correlation among the consecutive atomic score vectors, we generate a new sequence,

(3) $$X^{\left(k\right)}=\left(x_{1}^{\left(k\right)},x_{2}^{\left(k\right)},\cdots ,x_{T_{k}}^{\left(k\right)}\right),$$

which is a more general and smoothed form of $C^{\left(k\right)}$, as illustrated in Figure 3. Each $x_{t}^{\left(k\right)}\in R^{M}$ is an output of the vector valued function obtained using Time Varying Mean Vector (TVMV) [10], which computes $x_{t}^{\left(k\right)}$ or simply $x_{t}$ (that denotes the smoothed form of any instance of a composite activity) by processing all $a_{i}$ up to time *t*, denoted by $a_{1:t}$. This approach avoids the presumable disadvantages of directly using atomic scores. With this approach we first calculate $m_{t}=\frac{1}{t}\times \sum_{\tau =1}^{t}a_{\tau }$, where $m_{t}$ denotes the vector containing mean scores at time *t*. This is how the temporally local dependency on atomic scores obtained during the consecutive time intervals is maintained. Then we compute a unit-mean appearance vector $x_{t}$ to capture the direction at time *t*, $x_{t}=\frac{m_{t}}{∥m_{t}∥}$. Our ranking function Ψ, which is described in the following paragraphs, learns the evolution of this normalized vector over time. The way the TVMV is constructed, the dynamics of a composite activity in the forward flow with respect to time can be captured. Additionally, we also capture the backward flow by computing the TVMV on the reverse flow, starting from the latest time points to the earliest. The results show that computing the flow in both directions improves the recognition accuracy.

We focus on the relative ordering of atomic activities, that is, $x_{t+1}$ succeeds $x_{t}$, denoted as $x_{t}≺x_{t+1}$. As such we end up with the order constraint $O\left(X\right)=\left(x_{1}≺\cdots ≺x_{t}≺\cdots ≺x_{T}\right)$. We then learn to sequentially order the vectors $x_{t}$ (1≤t≤T) using a linear function $\Psi_{w}=\Psi \left(X;w\right)$ parametrized by w. Each instance $X^{\left(k\right)}$ of a composite activity is characterized by learning a different dynamics function $\Psi^{\left(k\right)}\left(X^{\left(k\right)};w^{\left(k\right)}\right)$ parametrized by $w^{\left(k\right)}$ [10]. In other words, $w^{\left(k\right)}$ characterizes the temporal evolution $D^{\left(k\right)}$ of $X^{\left(k\right)}$. As illustrated in Figure 3, we propose to use $w^{\left(k\right)}\in R^{M}$ of $\Psi^{\left(k\right)}$ as a representation of composite activity instance $X^{\left(k\right)}$.

In order to model the order constraints O(X), we can use the learning to rank paradigm in [11], also called ranking machines which optimizes a ranking function of the form $\Psi (t,x_{1:t};w)$ [10]. We can either choose a point-wise or a pair-wise ranking machine [11]. In rank pooling, the parameters w represent our composite activities. We implement a point-wise rank pooling approach by solving the constrained minimization problem, in which we seek $f(x_{t};w)\to t$ that is a direct mapping from the input vector $x_{t}$ to its time variable *t* based on the linear parameters w. In other words, we try to solve

(4) $$w^{*}=\underset{w}{arg min}\sum_{t}∥t-w^{T}.x_{t}∥,$$

where Support Vector Regression (SVR) is a robust approach to finding $w^{*}$. Thus, we can use SVR to encode temporal evolution *D* of composite activity *X*. Such a solution would also satisfy the order constraint $f\left(x_{i};w\right)<f\left(x_{j};w\right)$ if $x_{i}≺x_{j}$ due to the direct mapping of the form $f(x_{t};w)\to t$.

Real-life data exhibit complex patterns that are difficult to capture by a linear model, therefore, we use non-linear feature maps to manage the underlying non-linearity of *D*. Thus, we extend the linear ranking machine (SVR) by incorporating a non-linear transformation on each $x_{t}$ of *X*. We use two separate non-linear transformations. The first uses the Hellinger kernel defined as follows [37]:

(5) $$\mathrm{Hellinger}\left(x_{ij}\right)=\sqrt{\frac{x_{ij}}{\sum_{j=1}^{n}x_{ij}}},$$

where $x_{ij}$ denotes the *j*-th component of $x_{i}$. The second non-linear transformation is a modified version of the power transformation taken from [38]:

(6) $$\mathrm{Power}\left(x_{ij}\right)=x_{ij}^{\prime }=x_{ij}^{\lambda },\lambda \in R$$

Then we normalize the score by $\frac{x_{ij}^{\prime }}{\sum_{j=1}^{n}x_{ij}^{\prime }}$. As the value of each $x_{ij}$ is between 0 and 1, the original proposal for the power transformation $x_{ij}=x_{ij}^{\prime }=\frac{x_{ij}^{\lambda }-1}{\lambda }$, described in [38] converts all $x_{ij}$ into negative values which poorly maps the input vector $x_{t}$ to its time variable *t*, thus leading to poor results. The modified version not only avoids negative values, preserves the statistical distribution of the data but also improves the results.

Finally, we use $w^{*}$ as a feature vector to represent a composite activity for standard supervised learning method. We construct a linear SVM for each category of the composite activities using the training data and examine their performance on test data.

## 4. Experimental Results

In this section, we present the details of acquiring the CogAge dataset and discuss about the experimental settings and results on this dataset. We also exhibit a comparative analysis of our approach with the other popular or state-of-the-art methods.

### 4.1. Dataset Acquisition

We collected the CogAge dataset for atomic and composite activities using the following unobtrusive wearable devices like smart phone, smart watches [39] and smart glasses.

- LG G5 smartphone [40] placed in a subject’s front left pocket of the jeans, providing 5 different sensory modalities: 3-axis accelerometer, gravity sensor, gyroscope, linear accelerometer (all sampled at 200 Hz), and magnetometer (100 Hz). We use this device to capture the body movement.
- Huawei watch [41] placed on a subject’s left arm, providing two different sensory modalities: 3-axis accelerometer and gyroscope (both sampled at 100 Hz). This device is used to capture the movements of the hand.
- JINS MEME glasses [42] worn by the subjects, providing 3-dimensional data of accelerometer (sampled at 20 Hz) for our experiments, in order to capture the head movement.

Please see Appendix A for the details of eight sensor modalities in the above-mentioned three wearable devices, and the system layout to acquire data from those devices.

The data acquisition process of atomic activities targets 61 different activities involving 8 subjects who contributed to collect over 9700 instances. The data acquisition process for 7 composite activities was performed by 6 subjects using the same three wearable devices. An Android-based data acquisition application was developed to collect data for composite activities. The application connected Huawei watch and JINS MEME glasses to LG G5 smartphone via Bluetooth and saved the data of 8 sensory modalities locally on the memory of the smartphone. In this way, it became convenient for the subjects to move with the set of devices to their kitchens, washrooms, or living rooms, and perform the activities naturally. We collected over 1000 instances of composite activities. However, some of instances had missing sensory data and were removed from the dataset later. Therefore, we performed the experiments on 890 instances. Table 1 shows the list of composite activities and their counts. The length of each activity is not fixed as analogous to real-life events. It varies from 5 min to 30 s because some composite activities like preparing food take a long time to be completed, while there are some composite activities like handling medications, that take a shorter amount of time (although the lengths of composite activities were different, we did not use them as features for the classification because our goal is to recognize these activities in an online settings where we may not be able to determine their lengths in advance). The data collection for atomic and composite activities was carried out separately for training and testing phases on different days. We intended to include variations while performing the activities. The subjects who participated in data collection of atomic and composite activities come from different countries with diverse cultural backgrounds. Their ways of performing an activity were also varied. Therefore, we believe that the collected data includes a variety of activity executions that are suitable for examining the generality of our rank pooling method, despite the low number of subjects. Appendix A provides more description of data acquisition setup, sensor modalities, and the complete list of atomic activities.

### 4.2. Implementation Details of Activity Recognition

Sensory data of composite activities are firstly provided to the codebook-based atomic activity recognizer. The data transfer buffers (queues) are created for each type of sensors data. These buffers contain data of 1500 time points for accelerometer, gyroscope, linear accelerometer, and gravity sensors of smartphone. For smart phone magnetometer, and accelerometer and gyroscope of the Huawei watch, the buffers with 450 time points are used. A buffer with 150 time points is prepared for accelerometer of JINS glasses. Atomic activity recognition is performed every 2.5 s using sensor data temporally stored in the aforementioned buffers. It produces a series of vectors containing atomic scores of 61 atomic activities (i.e., $a_{i}^{\left(k\right)}$ in Section 3.2). Thus, we receive a matrix of atomic scores for each instance of a composite activity, where the number of rows depends upon the length of the composite activity, as depicted in Figure 3. For example, an activity with the length of one minute approximately produces 24 vectors of atomic scores. This matrix is considered as the characterization of a composite activity. Each row of the matrix measures the atomic scores within a time-window. Whereas each column provides atomic scores for a particular atomic activity in consecutive time-slices. The transitional patterns of these scores are important to recognize a composite activity. These kinds of matrices are approximated by the feature vectors using pooling techniques. We have constituted feature vectors for composite activities using several pooling techniques described in following sections.

#### 4.2.1. Max and Average Pooling

Max and average pooling are well known techniques to reduce the dimensionality of input data while keeping the characteristic information within the output. In our composite activity recognition problem we used these pooling techniques to transform a matrix of atomic scores into a feature vector. Max pooling is effective to get good accuracy and also guarantees that no important information in terms of atomic scores will be missed as the feature vector is constructed using the maximum score of each atomic activity during the execution of a composite activity. We have also used average pooling technique as it is more robust to mis-recognition as the whole spectrum of atomic scores over time effects the average value. Both pooling techniques yield 61-dimensional feature vectors for each instance of a composite activity. Despite their simplicity and effectiveness, they are inadequate to capture the temporal evolution of a composite activity.

#### 4.2.2. Rank Pooling

First of all, we validate our choice to use TVMVs in our rank pooling method. Table 2 shows a performance comparison between directly using atomic scores and using TVMVs. The results clearly show that ranking functions applied on TVMVs capture the temporal evolution of the composite activities better than when they are applied to atomic scores directly. Mainly due to the smoothness, the noise in atomic scores is removed and secondly the temporal transition captured in TVMVs makes it more correlated with time variables. Such a correlation is exploited by a ranking machine to learn the evolution of a composite activity over the time in the input data.

SVR is applied on TVMVs with soft-margin parameter C=1 and the parameters w and the bias of SVR are jointly used as the feature vectors of forward rank pooling. For reverse rank pooling we computed TVMVs in the reverse order and passed to SVR. In order to handle the non-linearity of input data, we used Hellinger and power transformations on TVMVs. The details of feature vectors based on these transformations, their notations and lengths are explained below:

- *Rank Pooling* refers to the 62-dimensional feature vector (FV) obtained after forward rank pooling. It contains a 61-dimensional weight vector w and the bias *b* of the SVR trained for each composite activity instance.
- $RP_{\mathrm{FwdRev}}$, is a 124-dimensional FV obtained by concatenating forward and reverse rank pooling outputs.
- $RP_{\mathrm{Hellinger}}$, is a 62-dimensional FV produced by applying Hellinger transformation on forward rank pooling FV.
- $RP_{\mathrm{Hellinger}\_\mathrm{FwdRev}}$, is a 124-dimensional FV obtained by applying Hellinger transformation on forward and reverse rank pooling FV.
- $RP_{\mathrm{Power}}$, is a 62-dimensional FV obtained by applying power transformation on forward rank pooling FV.
- $RP_{\mathrm{Power}\_\mathrm{FwdRev}}$, is a 124-dimensional FV obtained by applying Power transformation on forward and reverse rank pooling FV.
- $RP_{\mathrm{Hellinger}\_\mathrm{Power}}$, is a 124-dimensional FV obtained after concatenation of Hellinger and power transformation of forward rank pooling FV.
- $RP_{\mathrm{HellingerFwdRev}\_\mathrm{PowerFwdRev}}$, is a 248-dimensional FV constructed by the concatenation of Hellinger and power transformations of forward and reverse rank pooling FV.

#### 4.2.3. HMM and LSTM Pooling

In order to capture the temporal evolution of a composite activity, we can consider other popular algorithms for temporal data, such as Hidden Markov Models (HMM) and Long Short-Term Memory (LSTM).

We implemented HMM using *hmmlearn* library [43]. We built separate HMMs for each of the composite activities. Each of these HMMs has a left-to-right structure and takes as input a sequence of atomic scores. The HMMs were trained on two different data settings as described in the Section 4.3. The composite activity of a test example is recognized as the one, for which the corresponding HMM outputs the highest probabilities in comparison to the other HMMs. We performed our experiments on different parametric settings and found the best results with 3 states and 1000 iterations. As described in Section 1, a composite activity is not characterized by a specific structure of atomic activities. This may be a reason why we got better results with 3 states instead of a larger number of states.

We also performed our experiments using LSTM. The training data was provided in the settings as described in the Section 4.3. The input layer of our model accepts 61 atomic scores at each time point. These atomic scores are abstracted using two LSTM layers that have 18 and 10 units, respectively. The LSTM layers were followed by two dense layers with 10 units. We used Adam optimizer to optimize the parameters in our model. For a test instance, the outputs of the second dense layer form the feature vector [44] for an SVM to identify the composite activity.

### 4.3. Evaluation Protocol

The CogAge dataset was split into the training and testing data in the following two settings:

- In first setting, we used a holdout method to split CogAge dataset into the training and testing parts. The training part contained the data collected by three subjects (i.e., S1, S3, S6) which consists of 472 composite activity instances. The data of the remaining three subjects (i.e., S2, S4, S5) consisting of 418 activity instances, are used as the testing part. The data split was performed on the basis of two points. First, we intend to include different subjects on training and testing data so that the generalization power of our approach for unseen subjects can be demonstrated. Second, we want to divide CogAge dataset as evenly as possible.
- The second setting is leave-one-subject-out cross validation in which we repeatedly trained our models on the data of five subjects and tested them on the remaining one subject’s data. In this setting, every instance of an activity is tested for once and remained the part of training set for five times. It eliminates the bias of how the data is split and also reduces the variance of results.

For each of the seven composite activities, we trained linear support vector machines (SVM) as binary classifiers. The training data was used to train models and the performance was examined on test data. The performance of these SVMs to classify composite activities are evaluated using the average F1-score, which is an evaluation measure independent of class partition and already used in previous work of activity recognition [6,36,44]. An F1-score is the harmonic mean of precision (number of true positives divided by the total number of examples evaluated as positive) and recall (number of true positives divided by the total number of positive examples). We first compute the F1-score for each class in a one-vs-all fashion, and then take their mean value to get the average F1-score. We also evaluate our methods with two additional evaluation metrics to make the comparison with others easier: the overall accuracy (number of examples correctly classified divided by the total number of examples), and the Weighted F1-score (sum of F1-scores for all classes, weighted by the class proportion) [44]. In the following discussion, an average F1-score and weighted F1-score are abbreviated as AF1 and WF1, respectively. Finally, for readers interested in the performance of our codebook-based atomic activity recognition, please refer to the website of this paper and also our paper [44] showing that the codebook approach achieves a reasonable performance of activity recognition compared to several other methods including deep learning-based ones.

### 4.4. Results and Performance Evaluation

For composite activity recognition, rank pooling obtains better results in comparison to the other pooling techniques like max, average, HMM and LSTM pooling. Specifically, Table 3 shows the results on the holdout setting. It clearly shows that the non-linear rank pooling $RP_{\mathrm{HellingerFwdRev}\_\mathrm{PowerFwdRev}}$ outperforms all other pooling techniques in terms of all three measures and produces the best results. Table 4 shows the results of leave-one-subject-out cross validation, where another variation of non-linear rank pooling $RP_{\mathrm{Hellinger}\_\mathrm{Power}}$ produces the best AF1 and WF1 scores as compared to other techniques. Although max pooling is slightly better than non-linear rank pooling in terms of accuracy, we consider AF1 is a better measure because it is computed on the basis of classes while the accuracy is computed on the basis of instances. The accuracy may be biased on unbalanced datasets which is the case here as *Handling Medication* has significantly more associated examples than *Preparing Food*. In particular, a dominant class which is well recognized may increase the accuracy of the whole system without the latter necessarily performing well for less represented classes. In other words, the performance of $RP_{\mathrm{Hellinger}\_\mathrm{Power}}$ is more stable and higher for seven composite activities than max pooling.

Interestingly HMM and LSTM pooling poorly performed and there can be a couple of reasons: First, the lack of typical temporal structure of composite activities might lead to the low performance of HMM as it is useful when there exist such a typical temporal structure. The second reason can be due to the lack of large-scale dataset because the probabilistic approach (HMM) and model with many parameters (LSTM) need a much larger dataset than our rank pooling approach. The results conclude that our rank pooling approach improves the accuracy of composite activity recognition by capturing well temporal relations among underlying components (i.e., atomic activities) with a relatively small amount of data.

### 4.5. Fusion of Rank Pooling with Average and Max Pooling

In order to capture the diversity of input data (i.e., atomic scores), the fusion of different results may lead to a performance improvement, while the fusion of very similar results is unlikely to do so [45]. Since different pooling techniques have their own strengths, their fusion can be used to achieve improved results. We performed experiments while combining different variants of rank pooling with max and average pooling techniques. The results of these experiments are reported in Table 5 and Table 6 for the holdout and leave-one-subject-out cross validation settings, respectively. While comparing these results to those in Table 3 and Table 4, we observe 2 to 4% improvements in AF1, Accuracy, and WF1 scores. This result is quite convincing to conclude that the fusion of different results yields performance improvements. In the following section, we use the fusion of rank pooling with max and average pooling to discuss the performance of our approach in real-world settings.

## 5. Discussion for Real-World Settings

Our goal is to use the developed method for real-world settings, especially for monitoring ADLs of elderly people in a nursing home. Considering this, we first examine and discuss the performance of our method in an online setting. In addition, it may be burdensome for elderly people to wear all of LG G5 smartphone, HUAWEI watch, and JINS MEME glasses. Hence, we check how the performance of our method changes when using a subset of these devices.

### 5.1. Online-Recognition System

**Figure 4:**
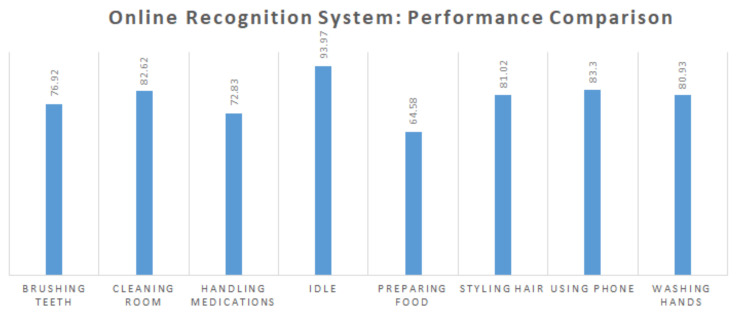
F1 scores for eight composite activities in the online setting.

**Figure A1:**
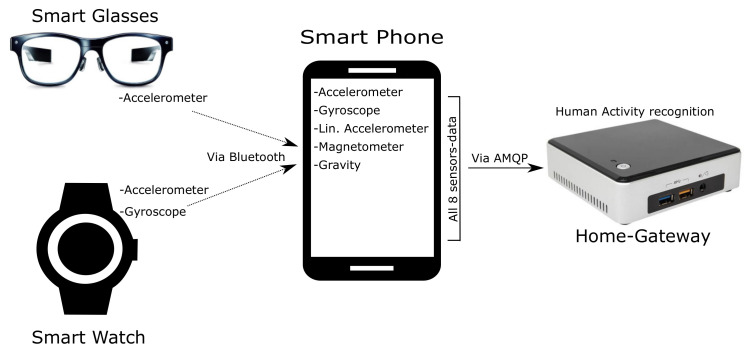
System layout: Our system uses three wearable devices. The sensory data from the smart glasses and the smart watch are firstly sent to smart phone via Bluetooth connection and then all sensory data is sent to home-gateway through Rabbit-MQ using Wi-Fi connection.

We implemented an online version of our recognition system which identifies composite activities in nearly real-time. A user wearing all three devices performs composite activities. The devices are connected according to Figure A1 and they send data to the home-gateway. Sensory data are received in the respective queues as described in Section 4.2. The online activity recognition is performed at every 2.5 s by encoding sensors data in the respective queues. The codebook-based atomic activity recognizer generates atomic scores which are further passed to the composite activity recognizer. In the online setting, we assume that a time window of 30 s is enough to recognize composite activities. We use the fusion approach that combines max pooling and average pooling with $RP_{\mathrm{HellingerFwdRev}\_\mathrm{PowerFwdRev}}$, based on our experiments where we found it more effective and robust than the other variations of rank pooling. Therefore, we use this approach to generate the feature vectors of composite activities to classify them using an SVM. The whole process of recognition takes less than 1 s. Figure 4 shows the results of our online recognition system, in which we display the F1 scores of seven composite activities along with another activity *Idle*. This activity represents the idle state of devices, i.e., when they are not worn by the subject and probably placed on a desk. In the online settings, we aim to log the duration of each composite activity performed by a user within a specific time period (one week or month) and generate a summary based on such logs. We found many time intervals where none of composite activities is performed and the devices might have been placed on a desk and remain unused. Thus, it is helpful and meaningful to categorize these time intervals as the idle activity class.

The demonstration videos of our atomic and composite activity recognition system can be found online (https://youtu.be/hr3i9I5Ga0M, https://youtu.be/J6WaO7jFOtU). Note that the composite activity videos were recorded using a previous version where we used linear rank pooling approach fused with max and average pooling. Thus, at some points, the accuracy remains a little low as compared to the version presented in this paper. Nevertheless, it can be observed that our system appropriately recognizes the user’s activities, and is robust to the changes of his postures.

### 5.2. Experiments on Different Device-Settings

We examine the performance of our composite activity recognition models on the following three different device settings:

- Phone Watch Glasses: we use all the three devices in the full version of our recognition system and all of the results shown in the above tables are also obtained using this device-setting.
- Phone Watch: in this device-setting, we use sensor values of LG 5S smartphone and Huawei watch to recognize our activities. This setting can be helpful for the users who want to use their own glasses or might not want to wear the glasses at all, because it hinders their activity execution (e.g., foggy glasses when cooking food).
- Watch-only: in this setting, we only use the accelerometer and gyroscope sensors of Huawei watch to recognize our activities. This is the lightest version of our system. Like for the glasses, a smartphone might not be convenient to a user for data recording, e.g., like if a user wants to use it instead of leaving it in their pocket.

**Figure 5:**
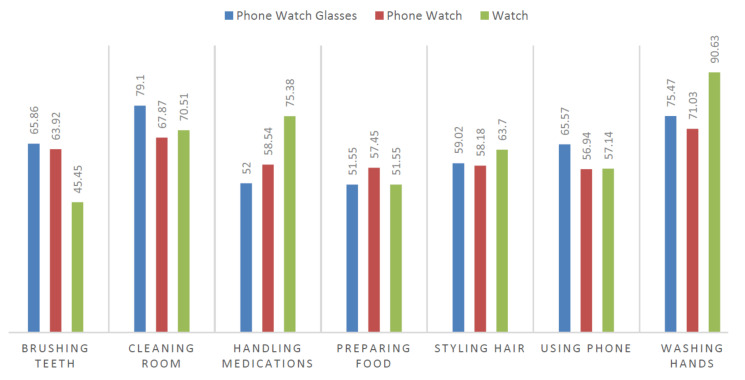
Performance comparison in terms of device combinations. The results are shown in terms of F1 scores.

We use the same layout as described in Figure A1. The sensory data are selected depending on a device setting. We find interesting facts that the best performances for the recognition of one activity are not necessarily obtained with all three devices combined, but sometimes with only the device placed on the part of the body actively involved in performing the activity. Figure 5 shows the performance comparison among the different device-settings. The statistics shown in this figure are acquired using the fusion approach on the holdout setting. Figure 5 clearly shows that *washing hands* can be effectively detected in the watch-only setting because hand movements provide enough information for distinguishing it from the other activities. On the other hand, *cleaning room* involves not only hands but also head and lower body movements (while bending, squatting, walking, etc.), so sensor data from all three devices increases the accuracy. In the future, if we consider more composite activities which involve movements of different body parts then we expect that using all devices should produce better results.

## 6. Conclusions and Future Work

In this paper, we proposed a two-level hierarchical model to recognize ADLs. The lower level recognizes 61 atomic activities using the codebook approach and outputs atomic scores for them over time. The higher level recognizes lengthy composite activities using the rank pooling approach applied to sequences of atomic scores. We conducted our experiments on the newly introduced CogAge dataset of wearable sensors data including 9029 examples of 61 different atomic activities and 890 examples of 7 different composite activities. We showed that our rank pooling approach outperforms the other well-known approaches like max pooling, average pooling, HMM, and discussed the possibility to use our approach in real-world settings. Although data for only 7 composite activities were used in the experiments, we believe that our model based on a large variety of underlying atomic activities is capable enough to recognize more composite activities like eating, bathing, getting dressed, toileting, and so on.

We implemented and tested a lighter version of our system on a few of elderly users’ household where composite activity recognition was being performed in the watch-only setting. This alternative version performed a little worse than the full version in terms of how correctly composite activities are identified. We also observed that our system performs better for young users compared to elderly people because of the slightly different way and speed to perform the same activity. Therefore, the recognition performance can be improved if we collect a few training examples from such an elderly user whose ADLs we are going to recognize later. This motivates us to introduce adaptivity in our system using a classifier feedback approach as our future work. This enhancement may lead to make our approach adaptable to get optimal results for users of all ages.

## Figures and Tables

**Table 1:** List of composite activities and the number of examples.

List of Composite Activities		
**Sr. No**	**Activity Name**	**Number of Examples**
1.	Brushing Teeth	128
2.	Cleaning Room	129
3.	Handling Medication	137
4.	Preparing Food	113
5.	Styling Hair	127
6.	Using Phone	128
7.	Washing Hands	128

**Table 2:** Comparison between direct use of atomic scores and their transformation into TVMVs. The evaluation measures are explained in Section 4.3.

Composite Activity Representation		
**Encoding Technique**	**Average F1**	**Accuracy**
Rank Pooling using atomic scores	31.50	35.07
Rank Pooling using TVMVs	48.74	51.49

**Table 3:** Comparison of pooling techniques: hold-out setting.

Composite Activity Recognition Results			
**Pooling Technique**	**Average F1**	**Accuracy**	**Weighted F1**
Rank Pooling	51.64	52.63	51.87
$RP_{FwdRev}$	55.99	56.94	55.89
$RP_{Hellinger}$	52.78	53.11	52.9
$RP_{Hellinger\_FwdRev}$	55.14	55.5	55.08
$RP_{Power}$	44.28	46.41	44.45
$RP_{Power\_FwdRev}$	55.17	55.5	55.12
$RP_{Hellinger\_Power}$	56.25	55.74	56.38
$RP_{HellingerFwdRev\_PowerFwdRev}$	60.91	61.48	60.95
Average Pooling	50.88	53.11	50.33
Max Pooling	55.26	57.42	54.6
LSTM (epochs=1000,#oflayers=4)	51.82	51.91	51.24
Hidden Markov Models (1000)	47.69	51.2	47.22

**Table 4:** Comparison of pooling techniques: leave-one-subject-out cross validation.

Composite Activity Recognition Results			
**Pooling Technique**	**Average F1**	**Accuracy**	**Weighted F1**
RankPooling	55.61	57.87	57.37
$RP_{FwdRev}$	50.59	53.39	51.89
$RP_{Hellinger}$	59.18	61.41	60.86
$RP_{Hellinger\_FwdRev}$	57.36	60.28	59.09
$RP_{Power}$	43.77	46.70	44.91
$RP_{Power\_FwdRev}$	46.78	51.04	48.67
$RP_{Hellinger\_Power}$	62.23	64.58	63.89
$RP_{HellingerFwdRev\_PowerFwdRev}$	60.70	62.91	62.23
Average Pooling	50.29	55.32	52.01
Max Pooling	60.74	64.79	62.41
LSTM Pooling	56.64	60.63	58.13
HMM Pooling	60.23	61.01	60.11

**Table 5:** Results of rank pooling fused with max and average pooling: hold-out setting.

Composite Activity Recognition Results: Pooling Techniques Combined			
**Pooling Technique**	**Average F1**	**Accuracy**	**Weighted F1**
RankPooling	58.57	58.37	58.43
$RP_{FwdRev}$	58.2	57.89	58.21
$RP_{Hellinger}$	59.59	59.09	59.56
$RP_{Hellinger\_FwdRev}$	58.80	58.85	58.63
$RP_{Power}$	57.91	58.13	57.48
$RP_{Power\_FwdRev}$	62.59	62.68	62.3
$RP_{Hellinger\_Power}$	61.96	61.48	62.00
$RP_{HellingerFwdRev\_PowerFwdRev}$	63.65	63.64	63.48

**Table 6:** Results of rank pooling fused with max and average pooling: leave-one-subject-out cross validation.

Composite Activity Recognition Results: Pooling Techniques Combined			
**Pooling Technique**	**Average F1**	**Accuracy**	**Weighted F1**
RankPooling	61.00	64.49	62.77
$RP_{FwdRev}$	59.29	63.18	61.00
$RP_{Hellinger}$	62.91	65.94	64.65
$RP_{Hellinger\_FwdRev}$	59.16	62.47	60.98
$RP_{Power}$	64.67	68.54	66.51
$RP_{Power\_FwdRev}$	64.39	68.65	66.24
$RP_{Hellinger\_Power}$	62.48	65.42	64.43
$RP_{HellingerFwdRev\_PowerFwdRev}$	62.34	65.24	64.12

## Supplementary Materials

The following are available online at https://www.mdpi.com/1424-8220/20/12/3463/s1.

## Abbreviations

The following abbreviations are used in this manuscript:

ADL	Activities of Daily Living
TVMV	Time Varying Mean Vector
HMM	Hidden Markov Models
LSTM	Long Short-Term Memory
RP	Rank Pooling

## Appendix A. Data Acquisition Setup

This appendix provides the details of sensor modalities collected for CogAge dataset. It also explains the data acquisition process of atomic activities.

### Appendix A.1. Wearable Devices and Sensor Modalities

Figure A1 shows the hardware configuration of our activity recognition system. The following three wearable devices are used to capture body, hand, and head movements:

I. LG G5 smartphone [40] is used to capture the body movement. It is placed in a subject’s front left pocket of the jeans, and provides 5 different sensory modalities:
  1. Accelerometer: This sensor produces a three-dimensional sequence that specifies acceleration forces (including gravity) acting on the smartphone’s x, y, and z axes. Its sampling rate is 200 Hz.
  2. Gyroscope: This sensor produces a three-dimensional sequence that presents angular velocities on the x, y, and z axes. Its sampling rate is also 200 Hz.
  3. Gravity: This sensor produces a three-dimensional sequence that represents gravity forces on the x, y, and z axes. Its sampling rate is also 200 Hz.
  4. Linear accelerometer: This sensor produces a three-dimensional sequence that specifies acceleration forces (excluding gravity) on the x, y, and z axes. It is also sampled at 200 Hz.
  5. Magnetometer: This sensor produces a three-dimensional sequence that describes intensities of the earth’s magnetic field along the x, y, and z axes. Such intensities are useful for determining the smartphone’s orientation. Its sampling rate is also 100 Hz.
II. Huawei watch [41] is used to capture the movements of the hand. It is placed on a subject’s left arm. It provides two different sensory modalities:
  6. Accelerometer: This sensor produces a three-dimensional sequence of acceleration forces applied to Huawei watch’s x, y, and z axes. Its sampling rate is 100 Hz.
  7. Gyroscope: This sensor produces a three-dimensional sequence of angular velocities on the watch’s x, y, and z axes. Its sampling rate is also 100 Hz.
III. JINS MEME glasses [42] is used to capture head movements of the subjects. It provides one sensor modality:
  8. Accelerometer: This sensor produces a three-dimensional sequence of acceleration forces on the glasses’ x, y, and z axes. It is sampled at 20 Hz.

We use the eight sensor modalities provided by these wearable devices. Figure A1 shows our system layout where sensors data from JINS MEME glasses and Huawei watch are initially sent to the LG G5 smart phone via Bluetooth connection and then all sensors data are further sent to a home-gateway through Rabbit-MQ using Wi-Fi connection [36]. Our home-gateway is established on Intel NUC NUC5i5RYK (CPU: Core i5-5250U 1.6 GHz, RAM: 16 GB, HDD: 450 GB, OS: Debian 4.8.4-1) where our atomic and composite activity recognition methods are executed.

### Appendix A.2. Data Acquisition of Atomic Activities

The data acquisition process of atomic activities targets 61 different activities involving 8 subjects who contributed to collect over 9700 instances. The data collection was carried out separately for training and testing phases on different days because we intended to include variations while performing atomic activities. In each phase the subjects were asked to wear the three devices and perform 10 examples of each activity where every example lasts for 5 s. After the removal of some examples where data were not appropriately recorded due to sensor errors, we use 9029 instances for our experiments. As shown in Table A1, 61 atomic activities are split into two distinct categories: 6 state activities characterizing the posture of a subject, and 55 behavioral activities characterizing his or her behavior. The reason is that a behavioral activity can be performed while being in a particular state, for instance, rubbing hands can be performed while either sitting, standing, or lying.

**Table A1:** List of state and behavioral atomic activities.

List of State Atomic Activities		
Standing	Sitting	Lying
Squatting	Walking	Bending
**List of Behavioral Atomic Activities**		
Sit down	Stand up	Lie down
Get up	Squat down	Stand up from squatting
Open door	Close door	Open drawer
Close drawer	Open small box	Close small box
Open big box	Close big box	Open lid by rotation
Close lid by rotation	Open other lid	Close other lid
Open bag	Take from floor	Put on floor
Bring	Put on high position	Take from high position
Take out	Eat small thing	Drink
Scoop and put	Plug in	Unplug
Rotate	Throw out	Hang
Unhang	Wear jacket	Take off jacket
Read	Write	Type
Talk using telephone	Touch smartphone screen	Open tap water
Close tap water	Put from tap water	Put from bottle
Throw out water	Gargle	Rub hands
Dry off hands by shake	Dry off hands	Press from top
Press by grasp	Press switch/button	Clean surface
Clean floor		
