# Supplementary material for: Rank Pooling Approach for Wearable Sensor-Based ADLs Recognition
Source: Sensors (Basel). 2020 Jun 19;20(12):3463. doi: 10.3390/s20123463 (PMC7349219; doi:10.3390/s20123463)
Supplement: Supplementary file 1 [file sensors-20-03463-s001.pdf]

sensors

MDPI

*Supplementary*

# Rank Pooling Approach for Wearable Sensor-based ADLs Recognition

We have uploaded all supplementary material including our datasets, codes, results, confusion matrices, detailed description of atomic activity recognition on the following url:

https://www.info.kindai.ac.jp/~shirahama/rank_pooling

User: rp

Pass: Atomic_Composite
